# The Quality of Salted Sun-Dried Meat from Young Nellore Bulls Fed Diets with Lauric Acid

**DOI:** 10.3390/foods11233764

**Published:** 2022-11-23

**Authors:** Sergiane A. de Araújo, Fernanda M. dos Santos, Rebeca D. X. Ribeiro, Analívia M. Barbosa, Ederson A. de Andrade, Gercino F. Virginio Júnior, Neiri J. A. dos Santos, Jarbas M. da Silva Júnior, Elzânia S. Pereira, Leilson R. Bezerra, Ronaldo Lopes Oliveira

**Affiliations:** 1 Pharmacy Department, Federal University of Bahia, Avenida Adhemar de Barros, 500, Salvador 40170110, Bahia, Brazil; 2 Animal Science Department, Federal University of Bahia, Avenida Adhemar de Barros, 500, Salvador 40170110, Bahia, Brazil; 3 Animal Science Department, Federal University of Ceará, Fortaleza 60021970, Ceará, Brazil; 4 Animal Science Department, Federal University of Campina Grande, Avenida Universitária, s/n–Jatobá, Patos 58708110, Paraíba, Brazil

**Keywords:** fatty acid, Nellore cattle, processed meat, salted sun-dried meat

## Abstract

This study aims to evaluate the quality of salted sun-dried meat from young bulls (Nellore cattle) fed with a diet containing 0.0, 0.5, 1.0 and 1.5% of lauric acid in the total dry matter (DM). Thirty-two Nellore bulls with initial body weight of 368 ± 32 kg were used. A linear decrease (*p* < 0.05) in pH and protein content of the salted sun-dried meat was observed with the inclusion of lauric acid. The moisture, ash, lipid, collagen content, water-holding capacity, cooking loss, color indexes (L*, a*, b*, C*), and shear force were not affected. Lipid oxidation at 7 days of storage increased linearly in the salted sun-dried meat. Most of the fatty acid composition of the salted sun-dried meat from the semimembranosus muscle of young bulls was not influenced (*p* > 0.05) by the lauric acid inclusion in the bulls’ diet. However, there was a linear increase (*p* < 0.05) in the SFA lauric acid (C12:0), PUFAn-3 EPA (C20:5*n* − 3) and DHA (C22:6*n* − 3), and a quadratic increase in the PUFAn-6 arachidonic (C20:4*n* − 6) due to lauric acid addition from palm kernel oil in the diet. There was a liner increase (*p* < 0.05) in the total ∑PUFA, ∑*n* − 6, ∑*n* − 3 contents of salted sun-dried meat from the semimembranosus muscle of young bulls and the h:H health index of the level of lauric acid inclusion in bull’s diet. In contrast, the thrombogenicity health index (TI) and ∑*n* − 6:∑*n* − 3 ratio content in salted sun-dried meat from the semimembranosus muscle of young bulls presented a linear decrease (*p* < 0.05) due to lauric acid addition in the bulls’ diet. Lauric acid (C12:0) inclusion up to 1.5% in the diet of young Nellore bull improved the fatty acid composition of the salted sun-dried meat, increasing EPA, DHA, *n* − 6 and *n* − 3, TI, and h:H indexes, which are associated with a better lipid quality of meat products, and further improves tenderness at the highest concentration.

## 1. Introduction

Meat processing aims to add value to meat by developing new products with different sensory characteristics, increasing shelf life and facilitating the transport and storage of products [1]. Simple technologies have given rise to new meat manufactured products, such as salted sun-dried meat [2]. Then, processing helps to improve meat quality and increase its shelf-life, and it also encourages the variety and availability of meat, rendering it and its products easier to handle, package, distribute, and market [1,2]. The salt used in the product preparation process partially dehydrates the meat, has an antibacterial effect and is responsible for conferring sensory characteristics with great acceptance by the consumer [2,3].

The growth in demand for food with sensory characteristics and nutritional quality to the consumer’s health has motivated an increase in developing a nutritional strategy to value ruminant products, such as the use of vegetal oils and plant secondary compounds [4,5,6,7,8]. Fatty acids (FA), the basic units of lipids, participate in enzymatic and regulatory pathways, energy storage and structural functions, and are important factors in the acceptance of meat products, influencing flavor, juiciness, tenderness, and shelf life [9,10]. The manipulation of the diet and consequently of the rumen environment can modify the lipid composition of the meat, altering the fatty acid profile [4]. Nutritional guidelines generally recommend reducing ruminant meat intake primarily due to its high saturated fatty acid (SFA) content, and its consumption above moderate amounts has been associated with an increase in chronic and coronary diseases and premature mortality [11,12,13].

In this sense, vegetal oils can be used to increase the energy density of diets, manipulate rumen fermentation, influence the absorption of nutrients and impact the characteristics and quality of the meat [7,8]. Palm kernel oil is a byproduct obtained from the palm fruit (*Elaeis guineenses*), which contains a large amount of lauric acid (C:12), and its effect on animal performance, digestibility, and fermentation parameters has been studied with the inclusion of up to 5.2% of dry matter of dietary total [7,8]. Lauric acid acts by destabilizing the cell membrane and interfering with energy metabolism and nutrient transport, leading to microbial cell death, mainly cellulolytic bacteria and ciliated protozoa [14,15,16].

The inclusion of oils rich in SFA in the diet of ruminants, such as palm oil and coconut oil, with high concentrations of lauric acid [17,18], may influence various attributes related to final product quality and its FA content [19] and oxidation [20], consequently increasing the product’s shelf life [10]. The rumen microbiota can change the unsaturated FAs present in the rumen throughout the biohydrogenation process [21]. This metabolism results in the synthesis of different FAs that are later absorbed by the small intestine [21,22]. Thus, the FAs used in ruminant diets promote the biosynthesis of different FAs, which are beneficial to human health, such the eicosapentaenoic fatty acid (EPA), docosapentaenoic acid (DHA), and conjugated linoleic acid (CLA), the latter having an anticarcinogenic effect [10,20,23,24].

In this context, this study tests the hypothesis that the inclusion of lauric acid obtained from palm kernel oil as a source of SFAs could reduce the fat oxidation of salted sun-dried meat without influencing the physicochemical composition and sensory attributes.

## 2. Materials and Methods

The research followed all the bioethical rules and guidelines applicable to animal studies described by the institutional Animal Ethics Committee. This trial was conducted at the Federal University of Bahia, which also approved animals in the study by their Ethics Committee for the Use of Animal in Experiments (UFBA Number of Approval: January 2015).

### 2.1. Animals’ Description and the Experimental Design Used

Thirty-two young bulls (Nellore, *Bos taurus indicus*) were used. Their initial body weight was 368 ± 32 kg and an average age of 24 months (730 days). The animals were marked with plastic earrings for better identification, vaccinated (clostridiosis), and de-wormed with Ivermectin (Ranger L.A. 3.5%).

The experimental animals were allocated in individual 2 × 4 m pens, with concrete floors, partially covered, containing feeders and drinking fountains. The animals were randomly distributed in a completely randomized design consisting of four treatments and eight experimental units (animals) per treatment. The experimental treatments consisted of diets with 0.0, 0.5, 1.0, and 1.5% inclusion of lauric acid in the diet’s total dry matter (DM). We used Palm kernel oil as the source of the FA, lauric acid, in the diet. The diets were formulated to be isoproteic and calculated according to the requirements recommended by the National Research Council [25] for finishing bulls. Tifton hay (*Cynodon* sp.) was used as roughage and ground to a size of approximately 5 cm, at a proportion of 40%, and mixed with 60% concentrate, composed of soybean meal, corn bran, mineral premix, and urea (Table 1). The experiment lasted 90 days, with 15 days for the animals’ adaptation to the facilities, management, and diets.

### 2.2. Diet Composition

Ingredient samples were collected and conserved at −18 °C, using polyethylene bags as a container, for further analysis of the chemical-bromatological composition of the experimental diets. Samples of ingredients and experimental diets were pre-dried in a forced ventilation oven at 55 °C for 72 h. Subsequently, the samples were crushed in Willey knife mills (Tecnal, Piracicaba, São Paulo, Brazil) with a 1 mm sieve, stored in plastic bottles with lids, labeled and subjected to laboratory analysis.

The analyzes were performed according to the procedures of the AOAC [26] for dry matter (DM, Method 967.03), ash (Method 942.05), crude protein (CP, Method 981.10), and ether extract (EE, Method 920.29) contents. Neutral detergent fiber content was evaluated following the methodology of Van Soest et al. [27]. Non-fibrous carbohydrates were calculated according to Mertens [28].

### 2.3. Slaughter Methods, Muscle Processing, and Meat Analysis

After the trial period, the animals were transported to a commercial slaughterhouse. After a 16 h food fasting period, the animals were stunned by a captive dart and subsequently bled, skinned, eviscerated, underwent carcass division longitudinally, weighed, and sent to a cold room where they remained under refrigeration at 4 °C for 24 h. pH measurement was performed after 24 h of cooling (pH final) in the center of the muscle (*Semimenbranosus*) using a Mettler M1120× pH meter (Mettler-Toledo International Inc, Columbus, OH, USA). While still in the slaughterhouse, the *semimembranosus* muscle was removed from each half of the carcass for further salted sun-dried meat production and analysis. The samples were identified, packed in plastic containers, and frozen (−20 °C) for analysis.

### 2.4. Production of Salted Sun-Dried Meat

The salted sun-dried meat confection process followed the Gouvêa and Gouvêa [2] recommendations, using 5% sodium chloride (refined table salt) (±150 g) per weight of the *semimembranosus* muscle (±3 kg). Excess fat was initially removed from the cuts for salting, which were later identified and packed in plastic containers. Then, an average of 5 cuts were made in the muscle for the subsequent application of sodium chloride. The cuts had the function of facilitating the penetration of salt in the meat. The salting period was 16 h, with the cuts kept under a controlled temperature at 25 °C. After salting, excess salt was removed in running water, and the meats were placed individually on metal hooks to remove excess water at room temperature (25 °C), remaining in this stage for another 8 h. Thus, after 24 h, the salted sun-dried meat was packed in plastic bags, identified, and frozen (−20 °C) for further analysis.

### 2.5. Physicochemical Analyses of Salted Sun-Dried Meat

Moisture, crude protein, ash, lipid and collagen contents of the salted sun-dried meat were measured by near infrared FoodScan equipment (FOSS Analytical A/S, Hillerod, Denmark), according to the methodology of the American Meat Science Association [29]. The pH was measured after the preparation of salted sun-dried meat using a hand-held digital pH meter (HI99163; HANNA Instruments, São Paulo, Brasil). The pH meter was calibrated using buffer solution (pH 4.01 and 7.0).

The analyses of water-holding capacity (WHC) were performed in triplicate to improve accuracy and followed the pressure method [30]. Two grams of salted sun-dried meat were placed on a circular filter paper, between two acrylic plates, then a force equivalent to 10 kg was applied to the top of the paper for approximately 5 min. The difference between the weight before and after the process was calculated. The quantity of water loss observed in the sample was expressed as a percentage of water exuded by the sample in proportion to the samples’ initial weight.

Cooking loss (CL) of salted sun-dried meat was determined according to American Meat Science Association [29]. Two samples (2.5 cm thick for each sample) free of subcutaneous fat were weighed before and after cooking. The samples were cooked on a grill (George Foreman Jumbo Grill GBZ6BW, Rio de Janeiro, Brazil). Each sample contained a stainless-steel thermocouple (Gulterm 700; Gulton of Brazil) positioned in the center (geometric) to monitor the temperature. When the temperature reached 71 °C, the samples were removed and exposed to room temperature until stabilized. The difference between the weight before and after cooking was used to determine the percentage of CL.

Shear force was used to evaluate the texture of salted sun-dried meat. Samples of the salted sun-dried meat were cut into cubes (following the longitudinal directions of the muscle fibers) with a diameter of approximately 1.0 cm, and the analysis was performed in triplicate. A texture Analyzer TX-TX2 device (Stable Micro Systems Ltd., Godalming, UK) was used at a 200 mm/min speed using a standard 1.016 mm shear blade and a Warner–Bratzler type blade 3.05 mm. The force used to cut was recorded and expressed in Newton force (N) [31]. The water activity analysis (Aw) was performed using AquaLab series 4TE equipment from the company Aqualab^®^, which analyzes the water activity by dew point with internal control of the sample temperature. The observed result was made available through the equipment monitor, and with this data, the averages were calculated.

The color analysis was conducted in a cross-section of muscle after thawing. The samples were exposed in a laboratory room to atmospheric air for 30 min to oxygenate myoglobin before obtaining readings through the colorimeter. The coordinates analyzed were the lightness (L*), redness (a*), and yellowness (b*). This analysis included a measurement performed at three different points in the studied muscle, and the mean was calculated for each coordinate per animal, as described by Miltenburg et al. [32]. The colors of salted sun-dried meat were measured using a colorimeter Minolta CR-400 (Konica Minolta, Tokyo, Japan) with 2° observer angle and 8 mm aperture diameter. The saturation index (Chroma, C*) was calculated using the equation described by Hunt and King [33]

*C = (a*^2^ + b*^2^)^0.5^ (1)

The concentrations of thiobarbituric acid reactive substances (TBARS) were measured to assess the extent of lipid oxidation [34] at 0, 7, 14 and 21 days after the manufacture of the salted sun-dried meat. The description of procedures, reagents and equipment used are detailed in Gesteira [35].

### 2.6. Fatty Acid Composition of the Salted Sun-Dried Meat

Fatty acid composition was analyzed according to O’Fallon et al. [36], with adaptations. The methodology applied for extracting fatty acids from the samples (sample preparation, materials, solutions, reagent, descriptions of the water bath, mixing, centrifugation, and removal of the supernatant) is described in detail by Macedo et al. [37]. A vortex (Fisatom 772, São Paulo, SP, Brazil) and a centrifuge (Centribio 80–2B, Equipar Ltd., Curitiba, PR, Brazil) were used in this step. The supernatant was collected and placed in a GC vial.

The separation and detection of FA were carried out using a Focus CG-Finnigan gas chromatograph with flame ionization detector, capillary column CP-Sil 88 (Varian), 100 m length, 0.25 mm in internal diameter, and 0.20 mm thickness. The carrier gas (hydrogen) was adjusted to a 1.8 mL/min flow rate. The details of oven heating temperature and time, carrier gas, injector temperature, detector temperature, and injection volume are described in detail by Macedo et al. [37].

Fatty acids were identified by comparing the retention times of the methyl esters in the samples with the Supelco^®^ TM Component Mix standards, using the C19:0 as internal standard (cat. 18919 Supelco, Bellefonte, PA, USA). Fatty acids were quantified by normalizing the areas of methyl esters, and g/100 g of the methyl esters of identified total FAs (FAME) were expressed. The sum (Σ) of the total SFAs, monounsaturated FAs (MUFAs), and polyunsaturated FAs (PUFAs) as well as the ΣPUFA:ΣSFA and *n* − 6:*n* − 3 ratios were calculated from the FA contents found in the experimental samples.

To assess the nutritional quality of the lipid fraction of the salted sun-dried meat, the atherogenicity index (AI) and the thrombogenicity index (TI) were calculated using the following equations:

AI = [(C12:0 + (4 × C14:0) + C16:0)]/(ΣMUFA + Σ*n* − 6 + Σ*n* − 3) (2)

TI = (C14:0 + C16:0 + C18:0)/[(0.5 × ΣMUFA) + (0.5 × Σ*n* − 6 + (3 × Σ*n* − 3) + (Σ*n* − 3/Σ*n* − 6)]. (3)

The ratio between the hypocholesterolemic and hypercholesterolemic (h:H) FAs was also calculated, as suggested by Ulbricht and Southgate [38]

h:H = (C18:1 *cis*-9 + C18:2*n* − 6 + C20:4*n* − 6 + C18:3*n* − 3 + C20:5*n* − 3)/(C14:0 + C16:0). (4)

We determined the desirable fatty acid (DFA) content following the recommendations of Rhee [39], wherein

DFA = (MUFA + PUFA + C18:0).(5)

The elongase and Δ9-desaturase enzymes activity indexes in 16 and 18 carbons FAs were determined following the recommendations of Smet et al. [40], using the equations:

Elongase = [(C18:0 + C18:1 *cis*-9)/(C16:0 + C16:1 + C18:0 + C18:1 *cis*-9)] × 100 (6)

-desaturase C16 = [C16:1 *cis*-9/(C16:0 + C16:1)] × 100 (7)

Δ9-desaturase C18 = [C18:1 *cis*-9/(C18:0 + C18:1 *cis*-9)] × 100. (8)

### 2.7. Sensory Attributes of the Salted Sun-Dried Meat

Salted sun-dried meat sensory evaluation was carried out according to the American Meat Science Association guidelines [29], using a panel of 80 consumers, 48 women and 32 men (aged between 18 to 54 years). Two salted sun-dried meat samples of each treatment were cut into cubes (approximately 3–5 g) and grilled on a preheated electric grill (George Foreman Jumbo Grill GBZ6BW, Rio de Janeiro, Brazil) at 170 °C until the temperature of the geometric center reached 71 °C. The samples were transferred to preheated beakers covered with aluminum foil and kept in a water bath at 75 °C so that the temperature of the samples remained between 65 and 70 °C until their distribution among the tasters.

The sensory panel was conducted from 9:00 to 11:00 a.m. The panel used 10 individual cabins with 10 participants per session (8 sessions). Each taster received eight samples of salted sun-dried meat (four treatments in duplicate), randomly distributed and coded. Each individual cabin contained cracker and salt water for ingestion between samples, to cleanse any residual flavor between samples. The duration time of each session was approximately 15 min.

Sensory attributes were determined using a 9-point hedonic scale: 1: disliked extremely; 2: disliked very much; 3: disliked moderately; 4: disliked slightly; 5: indifferent; 6: liked slightly; 7: liked moderately; 8: liked very much; 9: liked extremely. The sensory attributes evaluated were flavor, tenderness, juiciness and overall acceptance.

### 2.8. Statistical Analysis

A completely randomized design with four treatments (lauric acid inclusion at levels 0.0, 0.5, 1.0, and 1.5% DM) and eight replicates was adopted. PROC GLM command from the SAS^®^ statistical package was used [41]. Polynomial contrasts were used to evaluate the linear and quadratic effects of lauric acid inclusion levels on the physicochemical characteristics, nutritional characteristics, and sensory attributes of the salted sun-dried meat.

The statistical model used was:

Y*_ij_* = *μ* + s*_i_* +e*_ij_*, (9)

where Y*_ij_* = the observed value, *μ* = the general mean, s*_i_* = the effect of the levels of the lauric acid (0.0, 0.5, 1.0 and 1.5% DM), and e*_ij_* = the effect of the experimental error. The experimental design used in the sensory analysis was a randomized block design, with the scores that each taster assigned to each sample (of each experimental group) as an experimental unit and the consumer as a blocking factor. Additionally, the mean values obtained from the physicochemical characteristics and nutritional characteristics were compared by Tukey’s test. Significance was considered when the value of *p* < 0.05.

For the sensory characteristics of the salted sun-dried meat samples, the data were analyzed from a MIXED procedure of SAS, considering each observation from each taster as a repeated measurement within tasters, assuming a first-order autoregressive covariance matrix. The homogeneity of variances was evaluated for each variable, and, when needed, models were adjusted to accommodate heterogeneous variances, according to Milliken and Johnson [42]. Thus, the individual standard errors of the means (SEM) are presented. Significance was stated when *p* ≤ 0.05.

## 3. Results

The final pH of the salted sun-dried meat from the *semimembranosus* muscle of young bulls linearly decreased (*p* = 0.009) when the level of lauric acid inclusion in the diet increased (Table 2). A comparison of the means test revealed that the inclusion of lauric acid above 1.0% of lauric acid in the total dry matter promoted a decrease (*p* < 0.05) in the pH of the salted sun-dried meat compared to the control treatment (0% of lauric acid in the total dry matter, DM).

The inclusion of palm kernel oil as a lauric acid source in the bull’s diet resulted in a linear decrease (*p* = 0.041) in the protein content of the sun-dried meat. The comparison of the means test revealed that the inclusion of 1.5% of lauric acid (DM) caused a lower (*p* < 0.05) protein content of the salted sun-dried meat compared to the control treatment. The moisture, ash, lipid, and collagen content were not influenced (*p* > 0.05). The water activity of the salted sun-dried meat from the *semimembranosus* muscle of the young bulls showed a quadratic effect (*p* = 0.028) due to the level of lauric acid inclusion. However, the WHC, cooking loss, shear force, and color index (L*, a*, b*, C*) of the salted sun-dried meat from *semimembranosus* muscle of young bulls were not influenced by the treatments (*p* > 0.05). Lipid oxidation (Table 3) increased linearly at 7 days of storage (*p* = 0.046) and was not influenced by lauric acid inclusion on days 14 and 21 (*p* > 0.05). The control showed lower (*p* < 0.05) TBARS values at 7 days of storage of the salted sun-dried meat compared to the inclusion of 1.5% lauric acid in the comparison of the means test.

Most of the FA composition (Table 3) of the salted sun-dried meat from the *semimembranosus* muscle of young bulls was not influenced by the lauric acid inclusion in the bulls’ diet (*p* > 0.05).

However, a linear increase in the SFA lauric acid (C12:0; *p* = 0.004), PUFA *n* − 3 EPA (C20:5*n* − 3; *p* < 0.01) and DHA (C22:6*n* − 3; *p* < 0.01) was observed, and a quadratic increase in PUFA *n* − 6 arachidonic (C20:4*n* − 6; *p* = 0.023) due to the lauric acid addition from palm kernel oil in the diet. The comparison of the means test revealed that the inclusion of 1.5% lauric acid (DM) caused a greater (*p* < 0.05) deposition of lauric acid, EPA, and DHA in the salted sun-dried meat compared to the control treatment.

There was a liner increase (*p* < 0.01) in the total ∑PUFA, ∑*n* − 6, ∑*n* − 3 contents of the salted sun-dried meat from the *semimembranosus* muscle of young bulls as well as the h:H health index by the level of lauric acid inclusion in bulls’ diet (Table 4). The total ∑SFA, ∑MUFA, ∑PUFA:∑SFA ratio, atherogenicity index (AI), and enzymatic activity were not influenced by the inclusion of lauric acid in the bulls’ diet (*p* > 0.05). However, thrombogenicity health index (TI) and ∑*n* − 6:∑*n* − 3 content ratio in the salted sun-dried meat from the *semimembranosus* muscle of young bulls presented a linear decrease (*p* < 0.01) due to the lauric acid addition in the bulls’ diet. The comparison of the means test revealed that the inclusion of 1.5% lauric acid (DM) caused a greater (*p* < 0.05) deposition of ∑PUFA, ∑*n* − 6, ∑*n* − 3, and h:H in the salted sun-dried meat compared to the control treatment. On the other hand, lower TI index values (*p* < 0.05) were observed in the 1.5% treatment compared to the control.

Lauric acid inclusion promoted a better evaluation by the consumers to the tenderness (*p* = 0.023) of the salted sun-dried meat of animals fed with 1.5%, comparing with other levels of inclusion (Table 5).

The overall acceptance also showed higher (*p* = 0.045) scores in the salted sun-dried meat of animals consuming 1.5% (7.45) lauric acid compared to bulls’ salted sun-dried meat fed with 1.0% (6.98), but it was statistically similar to the bulls’ salted sun-dried meat that were not fed (7.21), or that received 0.5% (7.24) of lauric acid.

## 4. Discussion

Palm kernel oil (PKO) is a byproduct rich in medium-chain fatty acids (MCFAs) and saturated fatty acids (SFAs), especially lauric acid [14,43]. In some regions, this byproduct can replace expensive lipid sources; however, there is a need to evaluate the benefits of its inclusion in the quality of meat ruminant and its products (e.g., burger, sausage, and sun-dried meat) [7,8].

The reduction in the protein content of salted sun-dried meat with 1.5% of lauric acid compared to control (0%) may be related to the difference in lipid content of the diets and the intake of nutrients [8]. Diet energy content can affect the chemical composition of sun-dried meat, mainly affecting fat deposition and influencing protein deposition [44,45]. Furthermore, some studies demonstrate that the inclusion of palm kernel oil for ruminants promotes a linear reduction in the consumption (kg/day) of DM and crude protein; on the other hand, it produces an increase in EE consumption [7,8]. The same reductions in dry matter consumption can be identified in Table 1 with an intake of 9.87, 9.78, 7.19, and 5.18 (kg/day) for diets 0, 0.5, 1.0, and 1.5% lauric acid level in total diet (DM), respectively.

The final pH values of the meat (5.5 mean) suggest that there was no pre-slaughter stress or diet effect and, therefore, no changes in the glycogen level [46,47]. The values found in this experiment are within the range considered normal for beef meat (5.4 and 5.8; [48,49]. Although there was a difference between the pH salted sun-dried meat (5.6–5.8), the values were within the standards (5.5–5.9) [35,45]. With the similarity and the standard pH found in the salted sun-dried meat samples from both treatments, no changes were observed in cooking losses, water holding capacity, and color [17,45]. The lack of effect of lauric acid inclusion on the WHC of the salted sun-dried meat is in agreement with Popova [17], who evaluated the inclusion of coconut oil in lamb’s meat, and with Fiorentini et al. [19], who considered beef meat from Nellore cattle fed with oils rich in SFAs and UFAs and also did not find an effect of oil inclusion on the WHC. The WHC is mainly influenced by pH values [17], which were similar and within the pattern in the present study. The processing to obtain salted sun-dried meat (curing, salting, freezing, and thawing) are factors that can also change the WRC of the meat [30], however without effect in the study due to equality on the production of sun-dried meat. Salt (NaCl) acts by solubilizing meat proteins, and this process contributes to the emulsification of fats [45]. Processing may have altered values, but the diet could not influence them. There are several reported results showing that the use of lauric acid in the diet does not affect cooking loss; Najafi et al. [50] and Francisco et al. [51] showed these results in goat and cattle meat under the inclusion of different oils in the diet, including 2% palm oil. The loss of water is directly related to WHC; the higher the WHC, the lower the water loss during physical processing [48,50].

The shear force of salted sun-dried meat was not influenced by the inclusion of lauric acid, obtaining average values between 18.3 (1.83 kgf/cm^2^) and 23.9 (2.39 kgf/cm^2^). Values up to 3.2 kgf/cm^2^ are considered extremely tender, while values above 4.5 kgf/cm^2^ are less tender [47,52]. Different research has reported similar results, where the inclusion of different oils did not affect shear force [20,50,51].

Color is the main sensory characteristic appreciated by the consumer. Its evaluation is linked to freshness and adequate processing [53]. Meat color can be influenced by feed, animal age, weight, and pH [53]. In this study, even with a slight difference in the pH, the inclusion of lauric acid did not influence the color index of the sun-dried meat. It should be noted that a natural reduction in the comparison of meat with salted sun-dried meat may occur due to the salting processes, which influences the transformation of the myoglobin present in meat into metmyoglobin [54]. Even though the color of the meat may become browner, salted sun-dried meat is known by consumers to have a different color from fresh meat, and its color, therefore, does not influence purchase decision [45,55].

Regarding the fat present in ruminant meat, the high concentration of SFA and the low concentrations of polyunsaturated fatty acids (PUFAs) have been reported, making the meat less attractive [56]. However, meat from ruminants can still be an alternative for consuming long-chain polyunsaturated fatty acids beneficial to human health [23]. The salted sun-dried meat of the animals fed with 1.5% lauric acid improved the concentrations of eicosapentaenoic fatty acid (EPA) and docosapentaenoic acid (DHA). DHA and EPA acids are long-chain polyunsaturated fatty acids that can benefit human health, although they are in small portions of beef [23]. These acids are responsible for reducing lipid blood concentrations and, consequently, for reducing the risk of cardiovascular diseases, in addition to protecting against mental problems in adults, chronic headaches, and attention deficit disorder.

Among the saturated fatty acids found in beef, 30% of the total is represented by stearic acid. Additionally, myristic and palmitic acid are considered the most important fatty acids, and myristic acid has a four times greater potential to increase cholesterol than palmitic fatty acid, although both are relevant because they are considered hypercholesterolemic [47,57]. In the sun-dried meat of the study, the amounts of palmitic, stearic, and meristic acids had no effect between treatments and represented 49.2, 41.8, and 4.8% of the total amount of SFAs, respectively. The inclusion of palm kernel oil caused an increase in lauric acid (C:12) in the diet (Table 1) with 3.16, 26.4, 28.5, and 36.2% FAME for treatments 0, 0.5, 1.0, and 1.5, respectively. There was greater availability for the animals, reflecting a higher deposition of these FAs in the salted sun-dried meat of the treatment with 1.5% compared to the control.

The inclusion of lauric acid did not affect CLA and stearic acid in sun-dried meat. CLA is found only in ruminant derivatives, and an increase in CLA in meat and meat derivatives is highly desirable due to its anti-cancer potential and ability to reduce the risk of diabetes and heart problems [12,56,58]. It is possible that lauric acid in the diet in both treatments has the effect of inhibiting the biohydrogenation process, and the high concentrations of PUFAs may have interfered with this process, resulting in intermediate FAs [22].

The experimental diets also improved ƩMUFA in sun-dried meat, consequently improving TI and h:H ratio due to the major *n* − 6 and *n* − 3. The Σ*n* − 6 and Σ*n* − 3 ratio parameter is related with cardiovascular diseases and obesity [3,45]; it also did not change from lauric acid inclusion, and, in our salted sun-dried meat, ranged from 6.52 at 5.21, which indicates that the proportion of Σ*n* − 6 is above the proportion of Σ*n* − 3. The lauric acid inclusion promoted a range of h:H between 1.77 and 1.85, and TI averages ranging between 1.56 and 1.39, respectively, indicating good indices only with lauric acid inclusion. According to Ulbricht and Southgate [38], the values recommended for meat and meat products range between 0.79 and 1.39 for TI.

The results regarding meat oxidation found throughout the storage period showed TBARS values below the limit of 2.0 mg/kg malonaldehyde, which is considered a limit for acceptable consumption [59]. Meat exposure during the production of salted sun-dried meat may have accelerated lipid oxidation. Effects on oxidation–reduction were also found by Castro et al. [20], after including oils with different fatty acid profiles.

Tenderness is directly influenced by the pH of the meat and its WHC [3,17], and the pH and water activity were influenced by the treatments, although they did not affect the WHC, cooking loss, or shear force, resulting in the meat being extremely tender. The tenderness attribute is also directly related to the succulence of meat, where the more tender the meat is, the more quickly the juices are released during chewing, generating a greater sensation of succulence [44,60]. Our results showed higher tenderness of salted sun-dried meat from the 1.5% lauric acid diet than the control. These results were not expected as pH and WSC were similar between treatments [3,17].

The measurement of lipid oxidation by the TBARS method does not allow us to affirm that there was no lipid or protein peroxidation completely, as there are several products and tests to quantify the degree of oxidation. Nevertheless, the AI and h:H scores are not considered exclusive assessments for atherogenic property, as in lipid quality, there are several factors associated with assessing the product’s risks and benefits for human consumption.

## 5. Conclusions

Lauric acid (C12:0) inclusion up to 1.5% in the diet of young bull Nellore improved the fatty acid composition of salted sun-dried meat, increasing EPA, DHA, *n* − 6 and *n* − 3, TI, and h:H indexes, which are associated with better lipid quality of meat and meat products, and further improves tenderness at the highest concentration. More studies should be carried out to evaluate the effect of lauric acid in the ruminant diet to complement these results and the use of complementary methods, which associate the lipid quality of salted sun-dried meat and the effects on the health and consumption of humans.

## Figures and Tables

**Table 1:** The chemical composition of the experimental diet’s ingredients.

Item	Lauric Acid Level ^1^ (% Total DM Diet)			
	0	0.5	1.0	1.5
Ingredient proportion (g/kg DM)				
Tifton-85 hay	400	400	400	400
Soybean meal	26.0	27.0	29.6	31.4
Ground corn	544	530	517	504
Palm kernel oil	0.0	11.5	23.0	34.6
Urea + ammonium sulfate ^2^	15.0	15.0	15.0	15.0
Mineral mixture ^3^	15.0	15.0	15.0	15.0
Chemical composition (g/kg DM)				
Dry matter (g/kg as fed)	905	906	907	908
Ash	66.3	66.2	66.2	66.1
Crude protein	124	124	124	124
Ether extract	32.6	43.4	54.1	65.0
Neutral detergent fiber_ap_ ^4^	363	361	359	358
Non-fibrous carbohydrate	442	432	423	414
NIDN (g/kg CP) ^5^	231	230	228	226
Acid detergent lignin	26.6	26.5	26.4	26.4
Cellulose	82.8	82.8	82.7	82.7
Hemicellulose	272	270	269	267
Total digestible nutrients	796	809	823	836
Fatty acids (% FAME)				
C12:0	3.16	26.4	28.5	36.2
C14:0	1.11	8.72	10.7	13.1
C16:0	30.0	20.4	18.6	15.4
C16:1 *cis*-9	0.11	0.14	0.16	0.05
C18:1 *cis*-9	42.9	28.3	26.5	22.2
C18:2 *cis*-9, *cis*-12	10.3	5.16	5.17	0.04
Animal performance				
Dry matter intake (kg/day)	9.87	9.78	7.19	5.18
Average daily gain (kg/day)	1.14	1.34	1.03	0.59
Slaughter body weight (kg)	512	533	502	468

^1^ Palm kernel oil is a source of the fatty acid: lauric acid; ^2^ Mixture of urea–ammonium sulfate used at a 9:1 ratio; ^3^ Guaranteed levels (kg product): Na: 178 g; Ca: 128 g; S: 12 g; P: 44 g; Mg: 5000 mg; Mn: 750 mg; Cu: 1250 mg; Co: 107 mg; I: 50 mg; Fe: 1400 mg; Zn: 3700 mg; Se: 12 mg; F: 440 mg; ^4^ Corrected for protein and ash; ^5^ Neutral detergent insoluble nitrogen.

**Table 2:** Physicochemical proprieties of the salted sun-dried meat from the *semimembranosus* muscle of young bulls (Nellore cattle) fed with different levels of lauric acid on their diet.

Physicochemical Variables	Lauric Acid Levels (% DM) ^1^				SEM ^2^	*p*-Value ^3^	
	0	0.5	1.0	1.5		Linear	Quadratic
pH meat (24 h)	5.51	5.47	5.45	5.46	0.07	0.661	0.752
pH salted sun-dried meat	5.84a	5.73ab	5.63b	5.68b	0.047	0.009	0.098
Moisture, g/100 g	70.2	70.7	70.0	70.1	0.324	0.457	0.523
Protein, g/100 g	21.2a	20.8ab	20.9ab	20.3b	0.251	0.041	0.740
Ash, g/100 g	6.34	6.18	6.55	7.08	0.395	0.158	0.395
Lipid, g/100 g	2.24	2.35	2.49	2.51	0.201	0.316	0.814
Collagen (%)	1.95	1.95	1.99	2.01	0.071	0.532	0.887
Water activity (AW)	0.87	0.87	0.88	0.86	0.006	0.533	0.028
WHC ^4^ (%)	28.8	26.2	27.7	27.1	0.454	0.141	0.058
Cooking loss (%)	27.0	30.1	28.5	25.9	3.103	0.714	0.369
Shear force (N)	18.3	23.9	22.1	21.9	1.96	0.435	0.177
Color index							
L* (lightness)	34.9	34.1	34.2	33.5	0.600	0.173	0.887
a* (redness)	19.2	17.9	18.4	18.6	0.972	0.747	0.461
b* (yellowness)	6.36	6.21	6.42	6.05	0.408	0.706	0.791
C* (saturation)	20.2	19.0	19.5	19.5	1.010	0.719	0.538
Lipid oxidation (TBARS)							
0 days	0.31	0.30	0.26	0.30	0.038	0.791	0.536
7 days	0.75b	0.79ab	0.86ab	0.97a	0.077	0.046	0.657
14 days	1.13	1.04	1.00	1.05	0.106	0.650	0.583
21 days	1.06	0.89	1.13	1.39	0.256	0.313	0.421

^1^ Palm kernel oil as the source of the lauric acid (fatty acid); ^2^ Standard error of the mean; ^3^ *p*-values: significant at 0.05, values with different letters among columns are different for *p* < 0.05; ^4^ Water holding capacity; values with different letters among columns are different for *p* < 0.05.

**Table 3:** Fatty acid composition (% FAME) of the salted sun-dried meat from the *semimembranosus* muscle of young bulls (Nellore cattle) fed diets with different lauric acid levels inclusion on their diet.

Fatty Acid (% Total Fatty Acids)		Lauric Acid Levels (% DM) ^1^				SEM ^2^	*p*-Value ^3^	
		0	0.5	1.0	1.5		Linear	Quadratic
Saturated fatty acids (SFA)								
Lauric	C12:0	0.05b	0.11ab	0.15ab	0.18a	0.024	0.004	0.622
Myristic	C14:0	2.20	2.61	2.33	2.09	0.524	0.783	0.501
Palmitic	C16:0	23.8	23.9	23.2	23.2	0.966	0.769	0.816
Margaric	C17:0	0.98	0.95	0.10	0.98	0.074	0.532	0.842
Stearic	C18:0	20.2	19.6	20.9	19.4	0.932	0.396	0.507
	Others	1.05	1.08	1.07	1.11	0.083	0.537	0.834
Monounsaturated fatty acids (MUFA)								
Myristoleic	C14:1 *cis*-9	0.48	0.43	0.49	0.51	0.05	0.432	0.532
Palmitoleic	16:1 *cis*-9	2.32	2.43	2.12	2.21	0.12	0.745	0.824
Oleic	C18:1 *cis*-9	36.9	35.3	37.1	35.2	2.461	0.535	0.681
	C18:1 *trans*-11	1.15	1.05	1.33	1.18	1.140	0.566	0.858
Eicosenoic	20:1 *cis*-9	0.16	0.15	0.16	0.2	0.02	0.342	0.723
	Others	0.08	0.10	0.09	0.08	0.004	0.934	0.911
Polyunsaturated fatty acids (PUFA) *n* − 6								
CLA	C18:2–*cis*-9. *trans*-11	0.12	0.12	0.24	0.18	0.12	0.226	0.626
Linoleic	18:2 *cis*-9. *cis*-12	6.19	5.72	5.77	7.97	6.19	0.403	0.354
γ-linolenic	C18:3*n* − 6	0.16	0.17	0.15	0.18	0.011	0.622	0.852
Arachidonic	C20:4*n* − 6	1.81	3.17	2.08	1.83	1.81	0.342	0.023
DTA	C22:4*n* − 6	0.79	0.98	0.81	1.17	0.220	0.337	0.720
	Others	0.15	0.17	0.17	0.12	0.011	0.521	0.102
PUFA *n* − *3*								
α-linolenic	C18:3*n* − 3	0.92	1.08	0.97	1.15	0.168	0.840	0.871
EPA	C20:5*n* − 3	0.24b	0.57ab	0.46ab	0.68a	0.045	<0.01	0.445
DHA	C22:6*n* − 3	0.04b	0.13ab	0.10ab	0.15a	0.054	<0.01	0.346
	Others	0.22	0.18	0.23	0.22	0.012	0.764	0.633

^1^ Palm kernel oil as the source of lauric acid; ^2^ Standard error of the mean; ^3^ *p*-values: significant at 0.05, values with different letters among columns are different for *p* < 0.05; CLA—conjugated linoleic acid; EPA—eicosapentaenoic acid; DTA—docosatetraenoic acid; DHA—docosahexaenoic acid; values with different letters among columns are different for *p* < 0.05.

**Table 4:** Means, sums, ratios, and nutraceutical compounds (% FAMEs) of the salted sun-dried meat from the *semimembranosus* muscle of young bulls (Nellore cattle) fed diets with different levels of lauric acid inclusion on their diet.

Variables	Lauric Acid Levels (% DM) ^1^				SEM ^2^	*p*-Value ^3^	
	0	0.5	1.0	1.5		Linear	Quadratic
Sums and ratios							
∑SFA	48.3	48.3	47.8	47.0	0.859	0.155	0.980
∑MUFA	41.1	39.5	41.3	39.4	1.543	0.706	0.610
∑PUFA	10.6b	12.3ab	11.0ab	13.7a	1.961	<0.01	0.536
∑PUFA:∑SFA	0.22	0.25	0.23	0.29	0.112	0.456	0.763
∑*n* − 6	9.22b	10.33ab	9.22b	11.45a	1.982	<0.01	0.224
∑*n* − 3	1.419b	1.965ab	1.761ab	2.199a	0.256	<0.01	0.645
∑*n* − 6:∑*n* − 3	6.50	5.26	5.24	5.21	1.143	<0.01	0.111
Health indexes							
Desirable fatty acids	71.9	71.4	73.2	72.4	2.326	0.109	0.211
h:H	1.77ab	1.73b	1.82ab	1.85a	0.092	<0.01	0.646
AI	0.63	0.67	0.63	0.60	0.066	0.944	0.835
TI	1.56a	1.49ab	1.51ab	1.39b	0.099	<0.01	0.112
Enzymatic activity							
Δ9-desaturase C16	8.88	9.23	8.37	8.70	0.114	0.823	0.853
Δ9-desaturase C18	64.6	64.3	64.0	64.5	0.312	0.713	0.512
Elongase	68.6	67.6	69.6	68.2	0.444	0.632	0.941

^1^ Palm kernel oil as the source of the fatty acid (lauric acid); ^2^ Standard error of the mean; ^3^ *p*-values: significant at 0.05, values with different letters among columns are different for *p* < 0.05; h:H, Hypocholesterolemic and hypercholesterolemic ratio of the fatty acids content; AI, Atherogenicity index; TI, Thrombogenicity index. Abbreviations: ∑SFA, Sum of saturated fatty acids; ∑MUFA, Sum of monounsaturated fatty acids; ∑PUFA, Sum of polyunsaturated fatty acids; ∑*n* − 3, Sum of omega-3 fatty acids; ∑*n* − 6, Sum of omega-6 fatty acids.

**Table 5:** Sensorial attributes of the salted sun-dried meat from the *semimembranosus* muscle of young bulls (Nellore cattle) fed diets with different inclusion levels of lauric acid on their diet.

Attributes	Lauric Acid Levels (% DM) ^1^				SEM ^2^	*p*-Value ^3^
	0	0.5	1.0	1.5		
Flavor	7.58	7.47	7.16	7.49	0.130	0.316
Tenderness	6.90bc	7.12b	6.72c	7.53a	0.159	0.023
Juiciness	7.22	7.20	6.89	7.26	0.152	0.423
Overall acceptance	7.21ab	7.24ab	6.98b	7.45a	0.140	0.045

^1^ Palm kernel oil is the fatty acid source; ^2^ Standard error of the mean; ^3^ Values with different letters among columns are different for *p* < 0.05.

## Data Availability

The data presented in this study are available on request from the corresponding author.
